# Struma ovarii with Hashimoto’s thyroiditis: case report and review of the literature

**DOI:** 10.3389/fmed.2024.1393083

**Published:** 2024-07-09

**Authors:** Huayun Tan, Tingting Zhang

**Affiliations:** Department of Obstetrics, Weifang People’s Hospital, Weifang, China

**Keywords:** struma ovarii, ovarian tumor, CA 125, malignant struma ovarii, Hashimoto’s thyroiditis

## Abstract

**Background:**

Struma ovarii, a rare ovarian neoplasm originating from germ cells within mature teratomas, typically manifests benign characteristics. However, instances of malignant transformation have been documented.

**Case description:**

This report discusses a 25-year-old woman who had surgery in May 2020 to remove teratomas from both ovaries. In 2023, an ultrasound showed a complex mass in her pelvis. Further imaging tests, including CT, MRI, and F-18 FDG PET/CT scans, along with high levels of the CA 125 protein, suggested a mass in her left ovary, initially thought to be ovarian cancer. However, a closer examination after surgery found thyroid tissue and several types of cell growth but no cancer, confirming the diagnosis of struma ovarii. The pathology of hypermetabolic thyroid nodules on F-18 FDG PET/CT confirmed Hashimoto’s thyroiditis.

**Conclusion:**

This case underscores the importance of considering struma ovarii in the differential diagnosis of ovarian masses, especially in patients with a history of teratomas. It highlights the challenges in distinguishing struma ovarii from ovarian cancer due to similar clinical signs and imaging. Struma ovarii can be associated with Hashimoto’s thyroiditis.

## Introduction

Struma ovarii is an uncommon tumor derived from germ cells within mature teratomas, mostly benign but can sometimes turn malignant (1). Diagnosing it before surgery is difficult, and it may cause hypothyroidism after removal.

Despite its rarity, understanding struma ovarii is crucial because of its potential for malignant change and the challenge in preoperative diagnosis (2). There’s a gap in knowledge about its behavior, especially concerning thyroid hormone production.

This study aims to provide insights from a case involving a 25-year-old female who underwent laparoscopic surgery to remove teratomas from both ovaries in May 2020. In 2023, she was found to have a complex pelvic mass on ultrasound, highlighting the importance of recognizing this condition for accurate diagnosis and effective management. We present the following case by the CARE reporting checklist.

## Case presentation

A 25-year-old woman sought medical attention for persistent lower abdominal distension over 3 months, experiencing intermittent abdominal pain. Ultrasound imaging revealed a mixed echogenic mass in the pelvic cavity, presumed to originate from the left adnexal region.

### Timeline

May 26, 2020: Laparoscopic debulking of bilateral ovarian teratomas with ovarioplasty, under general anesthesia.

June 15, 2023: Presentation with persistent lower abdominal distension and intermittent abdominal pain.

June 20, 2023: Second surgery to remove the suspected ovarian mass.

Her medical history was unremarkable for major trauma, blood transfusions, and allergies. Notably, her serum CA 125 level was elevated at 64.4 U/mL (normal range: 0.0–35 U/mL), while the levels of β-hCG (<0.200 IU/L, normal range: 0–5.3 IU/L), CA724 (3.89 U/mL, normal range: 0–6.9 U/mL), AFP (2.51 μg/L, normal range: 0–20 μg/L), CA199 (9.11 U/mL, normal range: 0–27 U/mL), and HE-4 (41.8 pmol/L, normal range: 1–140 pmol/L) remained within normal ranges.

**Figure 1:**
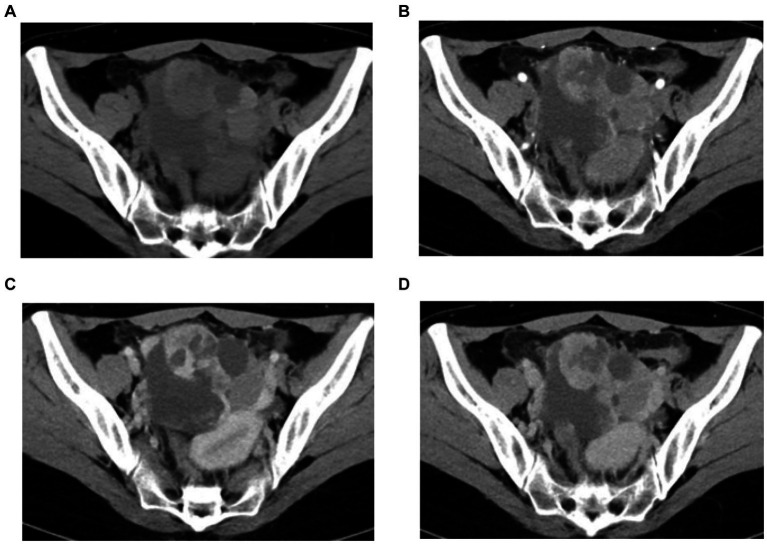
**(A–D)** Pelvic CT images. CT plain scan showed a left pelvic mass **(A)**, contrast-enhanced CT showed a cystic solid pelvic mass with heterogeneous enhancement in the peripheral areas and a non-enhanced central area in arterial phas **(B)**, venous phase **(C)**, and delayed phase **(D)**.

Abdominal ultrasound showed a mixed echogenic mass in the pelvis, CT scan showed a left pelvic mass measuring 8 cm × 7 cm, contrast-enhanced CT showed a cystic solid pelvic mass with heterogeneous enhancement in the peripheral areas and a non-enhanced central area. Mean plain CT value: 46 HU; arterial phase: 73 HU; venous phase: 107HU; delayed phase: 91 HU (Figures 1A–D).

**Figure 2:**
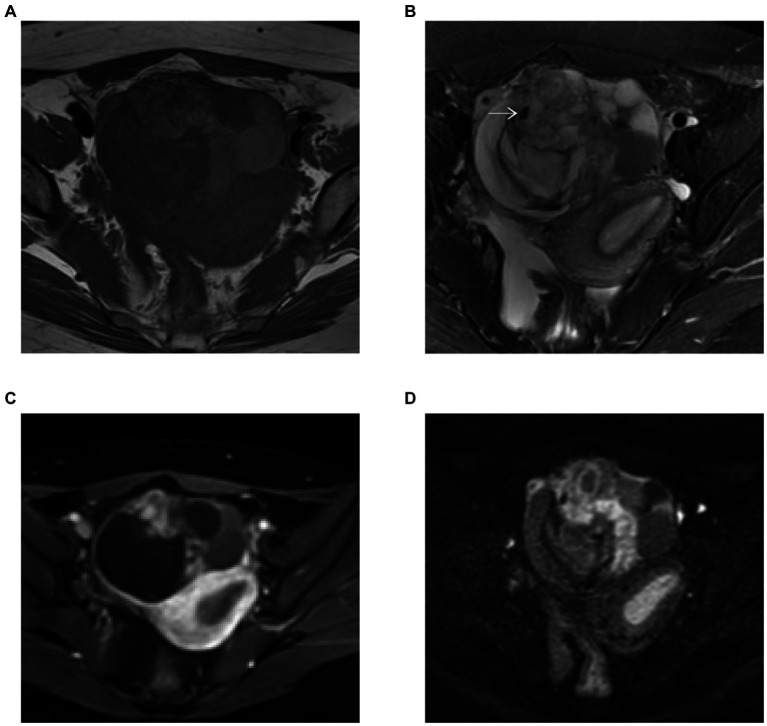
**(A–D)** Pelvis MR images. **(A)** T1-weighted image. MRI showed small foci of markedly low intensity (white arrow) on T2-weighted images **(B)** and contrast enhanced T1-weighted images demonstrated hyperenhancement within the solid component of the mass **(C)**. This area corresponded to hyperintensity on DWI (b = 800) **(D)**.

MRI showed small foci of markedly low intensity on T2-weighted images and contrast-enhanced T1-weighted images demonstrated hyperenhancement within the solid component of the mass. This area corresponded to hyperintensity on diffusion-weighted images (DWI) and hypointensity on apparent diffusion coefficient (ADC) maps. The restriction of diffusion to the solid component suggested the possibility of epithelial cancer (Figures 2A–D).

**Figure 3:**
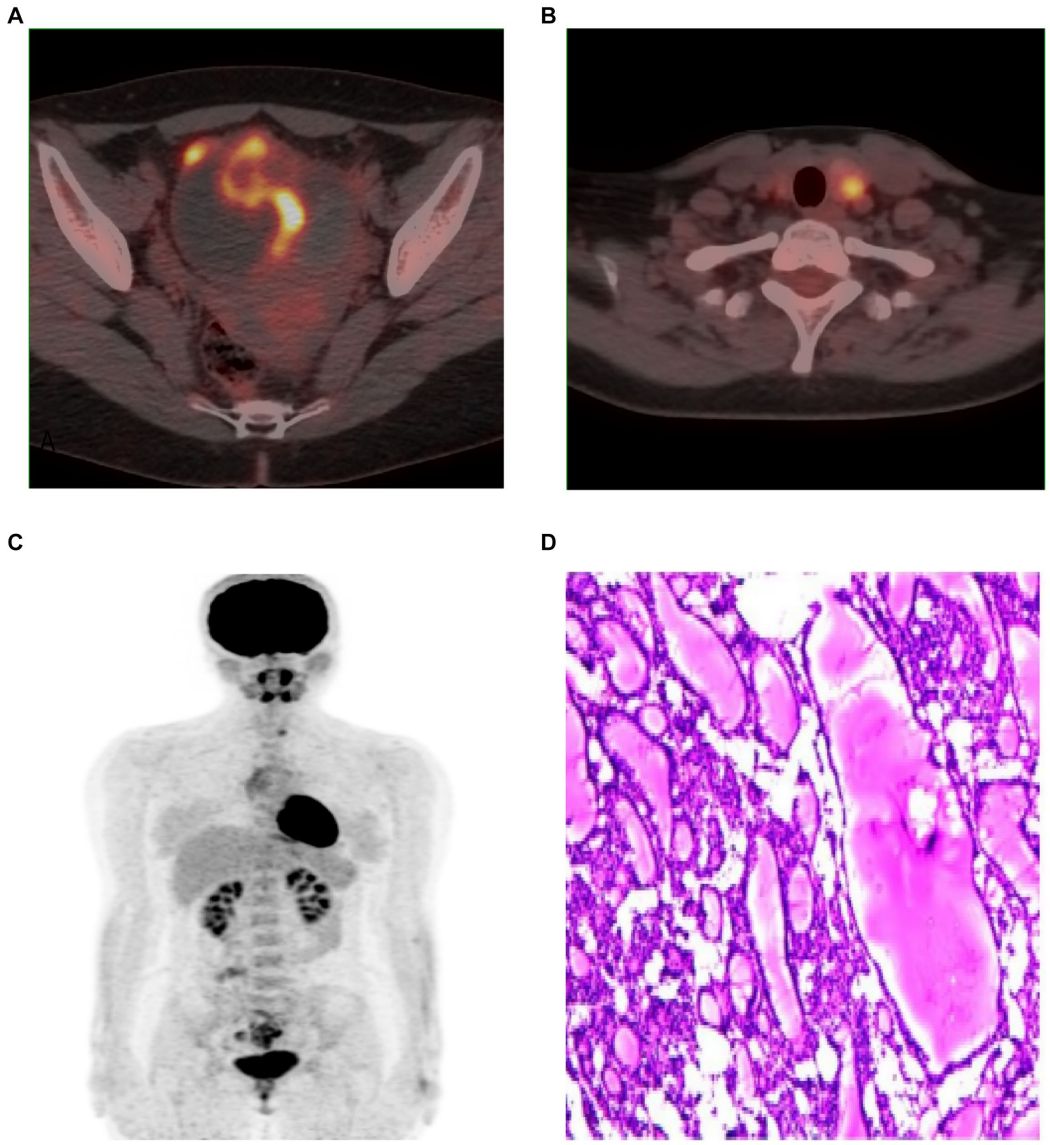
**(A–C)** F-18 FDG PET/CT images showed FDG uptake of pelvic tumor **(A)**, left lobe thyroid nodule **(B)**, MIP **(C)**. Microscopy showed a large amount of thyroid tissue, local follicular epithelial cell hyperplasia, consistent with struma ovarii **(D)**. (H and E, ×100).

Given the high CA 125 level and the suspicion of ovarian cancer, F-18 FDG PET/CT was used to further characterize the mass and to check for any hypermetabolic activity that might indicate malignancy or metastasis. FDG uptake in the solid component of the pelvis tumour (SUVmax = 6.8) was observed on F-18 FDG PET/CT, left lobe thyroid nodule SUVmax = 4.8 (Figures 3A–C).

However, it should be noted that while F-18 FDG PET/CT has high sensitivity, its specificity is low, making it difficult to distinguish between inflammation and tumors, necessitating pathological assessment for accurate diagnosis.

Considering the possibility of recurrence due to the previous laparoscopic ovarian tumor debulking, we opted for traditional surgical removal of the left ovary and tumor in the subsequent treatment to prevent recurrence. The postoperative pathological examination reported the presence of abundant thyroid tissue in the left ovary, with local follicular epithelial cell hyperplasia and adenomatous hyperplasia, consistent with a diagnosis of struma ovarii (Figure 3D). Additionally, luteal tissue and necrosis were observed. No malignant cells were detected.

Laboratory tests for all five thyroid functions were within normal limits: total triiodothyronine (TT3) was 2.2 nmol/L (normal range: 1.3–3.1 nmol/L), Total Thyroxine (TT4) was 94.9 nmol/L (normal range: 60–150 nmol/L), Free Triiodothyronine (FT3) was 5 pmol/L (normal range: 3.5–6.5 pmol/L), Free Thyroxine (FT4) was 14.6 pmol/L (normal range: 9–25 pmol/L), and Thyroid-Stimulating Hormone (TSH) was 0.6 mIU/L (normal range: 0.3–4.2 mIU/L).

The neck ultrasound revealed a nodule in the left lobe of the thyroid, classified as TI-RADS 3, indicating a probably benign nodule with a low risk of malignancy. A fine-needle aspiration biopsy was performed, and the nodule was assigned a Bethesda score consistent with Hashimoto’s thyroiditis (Bethesda Category II).

Despite F-18 FDG PET/CT not typically being the initial choice, the clinical suspicion of malignancy justified its use for staging, leading to the incidental discovery of a hypermetabolic nodule in the left thyroid lobe; although the ultrasound suggested a benign condition, a needle biopsy prompted by patient concerns confirmed Hashimoto’s thyroiditis, with follow-up pathology revealing lymph follicular hyperplasia and fibrosis.

The patient is currently recovering well post-surgery. The thyroid nodule in the left lobe, being asymptomatic, has not required any specific treatment and remains under continuous observation.

## Discussion

We reviewed clinical trials from 2000 to 2023, exploring the association between struma ovarii and thyroid nodules (Table 1) (2–14). Our focus was on the diagnostic challenges and management complexities arising from the coexistence of struma ovarii and thyroid disorders, particularly considering elevated CA125 levels are often mistaken for ovarian cancer indicators. Malignant transformation in struma ovarii, though rare, adds to diagnostic and therapeutic complexities.

**Table 1:** Clinical and diagnostic characteristics of struma ovarii cas.

Author and year	Clinical presentation	Lab OR imaging findings	Treatment and prognosis
Zhang et al. (2)	Recurrent malignant struma ovarii, hyperthyroidism, multiple metastases	Elevated CA125, Tg, FT4, TSH suppressed, CT images	Surgical intervention including liver mass dissection and omentectomy; diagnosis of malignant SO with metastases
Jin et al. (3)	Pseudo-Meigs syndrome	Elevated CA125, ovarian mass on CT	Surgical treatment; good prognosis
Yang et al. (4)	Malignant struma ovarii (Papillary carcinoma) with Hyperthyroidism	Hyperthyroidism, B-ultrasound	Surgical treatment; good prognosis
Loizzi et al. (5)	Pseudo-Meigs syndrome	Elevated CA125, ovarian mass on CT	Surgical removal; favorable prognosis
Ciccarelli et al. (6)	Thyrotoxicosis	Thyroid hormone levels, I123scintigraphy, CT and MR	Surgical and medical management; positive outcomes
Paladini et al. (7)	Mimics ovarian cancer, hyperthyroidism	Elevated CA125, abnormal thyroid function tests, I131scintigraphy, ultrasound	Accurate diagnosis and treatment; good prognosis
Hatami et al. (8)	Severe hyperthyroidism	Elevated thyroid hormones, MRI	Depends on malignancy extent; surgery and possible adjuvant therapy
Gild et al. (9)	Malignancy and hyperthyroidism symptoms	Thyroid function tests, thyroglobulin >3,000 μg/L	Radioactive iodine therapy; improved prognosis
Matsuda et al. (10)	Thyrotoxicosis	Elevated thyroid hormones	T3, T4, and thyroglobulin normalize within 2 weeks and TSH within 4–5 weeks after definitive surgery.
Fujiwara et al. (11)	Mimics ovarian cancer	Elevated CA125, I131 scintigraphy increased uptake and F18-FDG no uptake	Accurate diagnosis; generally good prognosis with surgery
Jung et al. (12)	Imaging findings may vary	Struma ovarii usually appears as a smooth marginated multicystic mass with a high attenuation lesion on precontrast CT	Surgical intervention; prognosis depends on timely diagnosis
Yamauchi et al. (13)	Imaging indicative of malignancy	CT, MR, PET/CT	Early detection and comprehensive treatment; favorable prognosis
Demir et al. (14)	Accurate localization of ectopic thyroid tissue	SPECT /CT showed struma ovarii uptake I131	Precise diagnosis and treatment; positive outcome

Struma ovarii is characteristically identified as a multicystic mass with smooth margins and solid components, exhibiting high attenuation on CT scans (15). The cystic features typically show low signal intensity on T1, and low intensity on T2-weighted MRI, and high density on unenhanced CT, indicative of a gelatinous colloid (2). The case under discussion, however, displayed atypical imaging features. Struma ovarii is usually benign, though malignant transformation is occasionally observed. Preoperative diagnosis is notably challenging, often confounding the selection of an appropriate surgical approach. Amongst well-differentiated neoplasms arising in struma ovarii, papillary thyroid carcinoma and strumal carcinoid are the most common, usually exhibiting minimal or non-aggressive behavior (3). Additionally, struma ovarii can show uptake of I-131 on SPECT imaging (7, 11, 14).

PET/CT is not routinely used in malignant struma ovarii (MSO), but may be helpful in cases of suspected malignancy or metastasis (12). Our investigation into struma ovarii, particularly within the context of thyroid nodules and its association with teratomas, underscores the rarity and diagnostic complexity of this condition. The case highlights the challenge of distinguishing struma ovarii from ovarian cancer due to overlapping imaging features and elevated CA125 levels, a common marker for ovarian malignancy. Notably, the presence of thyroid tissue and the histopathological features observed post-surgery were pivotal in achieving the correct diagnosis.

One of the strengths of this study is the comprehensive diagnostic approach, employing a range of imaging techniques (CT, MRI, PET/CT) and histopathological examination, which facilitated a detailed understanding of the tumor’s characteristics. However, the study is limited by its singular case design, which, while providing in-depth insights, may not fully represent the spectrum of struma ovarii presentations. Autoimmune thyroiditis with struma ovarii has been described in a few case reports (16). Since laboratory tests were normal for thyroid function, we recommend close follow-up to monitor thyroid function and ultrasound follow-up to assess the progression of Hashimoto’s thyroiditis.

Similar studies have also reported the benign nature of most struma ovarii cases, with a small fraction exhibiting malignant transformation (1). This case contributes to the body of evidence suggesting that struma ovarii, while typically benign, necessitates a careful diagnostic approach to differentiate it from more aggressive ovarian neoplasms. The preoperative diagnosis of struma ovarii is usually difficult. The tumor mimics a malignant tumor based on tumor marker assessments. Looking forward, improving the early detection of this tumor through new imaging methods and diagnostic markers is essential, aiming to better understand its nature and enhance treatment approaches for struma ovarii.

## Conclusion

The diagnosis of struma ovarii, often confused with ovarian cancer, is complex due to its unique presentation. This study highlights the role of advanced imaging and new markers in improving early detection and understanding of the condition. Struma ovarii can be associated with Hashimoto’s thyroiditis. Ongoing research and increased awareness are crucial for effective management of this rare tumor.

## Data availability statement

The raw data supporting the conclusions of this article will be made available by the authors, without undue reservation.

## Ethics statement

The studies involving humans were approved by the ethics committee/institutional review board of Weifang people’s hospital. The studies were conducted in accordance with the local legislation and institutional requirements. The participants provided their written informed consent to participate in this study. Written informed consent was obtained from the individual(s) for the publication of any potentially identifiable images or data included in this article. Written informed consent was obtained from the participant/patient(s) for the publication of this case report.

## Author contributions

HT: Investigation, Writing – original draft. TZ: Conceptualization, Investigation, Writing – review & editing.
